# Effects of intramuscularly injected plant-derived antimicrobials in the mouse model

**DOI:** 10.1038/s41598-022-09705-9

**Published:** 2022-04-08

**Authors:** Elizabeth J. Johnson, Jingyue Ellie Duan, Kanokwan Srirattana, Kumar Venkitanarayanan, Edan R. Tulman, Xiuchun Cindy Tian

**Affiliations:** 1 Department of Animal Science, University of Connecticut, 1390 Storrs Rd, Storrs, CT 06269-4163 USA; 2 Department of Pathobiology and Veterinary Science, University of Connecticut, Storrs, CT 06269 USA; 3 Present Address: Department of Animal Science, College of Agriculture and Life Sciences, Cornell University, Ithaca, NY 14853 USA; 4 Present Address: Embryo Technology and Stem Cell Research Center and School of Biotechnology, Suranaree University of Technology, Nakhon Ratchasima, 30000 Thailand

**Keywords:** Drug safety, Toxicology

## Abstract

With increasing antibiotic resistance, the use of plant derived antimicrobials (PDAs) has gained momentum. Here, we investigated the toxicity of *trans*-cinnamaldehyde, eugenol, and carvacrol after intramuscular injection in mice. Two doses of each PDA—300 and 500 mg/kg body weight—and vehicle controls were injected into the muscle of the right hind limb of CD-1 adult mice (n = 8/treatment). Ten physical/behavioral parameters were monitored hourly for 2 h and twice daily for 4 days post-injection together with postmortem examination of leg muscles and organs. Within the first 2 days of carvacrol treatment, one male died in each dose level and a third male receiving 500 mg/kg was removed from the study. No mortality was seen with any other treatment. Among all 81 parameters examined, significant higher relative liver weights (300 and 500 mg/kg eugenol groups; *P* < 0.05) and relative kidney weights (300 mg/kg carvacrol group; *P* < 0.001) were observed. Taken together, little to mild toxicity was seen for *trans*-cinnamaldehyde and eugenol, respectively, while carvacrol exerted more toxicity in males. This study lays the foundation for future extensive work with large sample size, varied treatment durations, and additional treatment levels.

## Introduction

Plants have been used as medicine for centuries^1^. Essential oils have been extracted from many different plants and possess anti-inflammatory, anti-microbial, and anti-tumor effects^2^. The active ingredients in essential oils, referred here as plant-derived antimicrobials (PDAs), are phenolic compounds such as carvacrol^3^, eugenol^4^, and *trans*-cinnamaldehyde^5^. While each PDA may have a different mechanism of action, they generally have antimicrobial effects through disturbance of the cytoplasmic membrane, disruption of proton motive force, electron flow and active transport, or coagulation of cell contents^6^.

With the increase of resistance to antibiotics, alternatives are needed to control pathogenic infection in both humans and animals^7^. Although essential oils and plant-derived antimicrobials have been studied for their growth-promoting, methane-inhibiting, and disease-preventing effects as feed additives in livestock^8–10^, no studies have been conducted to test the possibility of PDAs as an intramuscular injectable to treat infections.

In general, oral, intravenous (iv), intramuscular (im), and subcutaneous (sc) administration are possible routes for medication delivery. Effects and toxicity of PDAs have mostly been conducted by oral administration, sc or iv injections^11–13^. A very limited number of reports documented intraperitoneal (ip) injection of PDAs in the mouse model, mainly to explore their beneficial health effects. For example, eugenol oil ip injection exerted anti-nociceptive effects on acetic acid and formalin injected mice^14^. Furthermore, carvacrol ip injection reduced memory loss in a rat model of Parkinson’s disease^15^. While PDAs could be administered orally in large animals, such as domestic species, effective doses may be cost prohibitive. While parenteral administration of PDAs may be a viable alternative in livestock, im injection is likely more appropriate for reasons of convenience, cost, safety, and fast absorption.

The aim of this study was to determine if any local or systemic side effects in mice are elicited by im injection of PDAs (*trans*-cinnamaldehyde, eugenol, and carvacrol) at levels found to inhibit 100% bacterial growth in vitro (0.03% and 0.05%, equivalent to 300 and 500 mg/kg body weight). Here, we found a slight toxicity in the 500 mg/kg eugenol group, in which the relative liver weight (over that of the body weight) was significantly higher than that of the control group. No other statistically significant changes were seen in any other treatment group in any of the parameters measured. However, more acute toxicity in males than females was observed when injected with carvacrol. Local muscle relaxation effects were observed in all treatment groups. Overall, our results suggested *trans*-cinnamaldehyde and eugenol are generally safe at the locally injected levels, while carvacrol exerted more acute toxicity.

## Materials and methods

### Chemicals and reagents

Carvacrol (CAR), eugenol (EU), *trans*-cinnamaldehyde (TC), dimethyl sulfoxide (DMSO), and phosphate buffer saline (PBS) were purchased from Sigma-Aldrich (Sigma-Aldrich, Inc., St. Louis, MO, USA).

### Animals

A total of 72 CD-1 mice of 7–8 weeks of age, weighing 25–40 g, were obtained from Charles River Laboratory (Boston, MA, USA). Mice were housed in a biohazard level II AALAC-accredited facility, under 12:12-h (h) light–dark cycles in a temperature and humidity-controlled room. Mice had food and water ad libitum. Animal handling procedures were conducted in a Class II biosafety cabinet. Cabinet surface was decontaminated and sterilized with 10% bleach to prevent cross-contamination between consecutively handled mice. Experimental procedures were approved by the Institutional Animal Care and Use Committee (IACUC) of the University of Connecticut. All methods were performed in accordance with the relevant guidelines and regulations. The study is also reported in accordance with ARRIVE guidelines https://arriveguidelines.org).

### Experimental design

**Figure 1:**
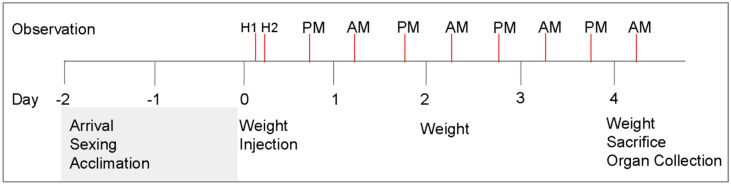
Animal treatment and observation schemes. Mice arrived on Day -2 and were sexed. The mice then had a 48 h acclimation time. On the morning of Day 0, the mice were weighed and injected. Mice were observed hourly for two h, and twice daily post-injection until Day 4 when mice were weighed and euthanized for dissection. Red and gray vertical bars indicate the times of observation and days of experimentation, respectively.

Experimental design is illustrated in Fig. 1. Briefly, mice were sexed upon arrival (Day − 2), separated into male and female cages, and given 48 h of acclimation time. The 72 mice were divided into nine groups of eight each (four females and four males) including three control groups and six treatment groups. PDA doses were based on in vitro studies and are shown in Table 1. PDA or control (DMSO) injections were given in the morning of Day 0. Mice were observed hourly for 2 h post-injection and twice daily for 4 days thereafter.

**Table 1:** Levels of PDAs used for treatments. DMSO was used as the vehicle control.

Treatments	Dose	N
DMSO (controls^a^)	450 mg/kg (6.34 mM) 750 mg/kg (10.56 mM) 900 mg/kg (12.68 mM)	8 (M = 4, F = 4) 8 (M = 4, F = 4) 8 (M = 4, F = 4)
TC	300 mg/kg (2.27 mM)	8 (M = 4, F = 4)
	500 mg/kg (3.78 mM)	8 (M = 4, F = 4)
CAR	300 mg/kg (2.0 mM)	8 (M = 4, F = 4)
	500 mg/kg (3.33 mM)	8 (M = 4, F = 4)
EU	300 mg/kg (1.83 mM)	8 (M = 4, F = 4)
	500 mg/kg (3.04 mM)	8 (M = 4, F = 4)

^a^Three different control groups were needed in order to control for the different concentrations of DMSO used in different PDA treatments. The controls and treated mice corresponded as follows: 10.56 mM (or 750 mg/kg) DMSO to control for 500 mg/kg TC and 500 mg/kg EU; 6.34 mM (or 450 mg/kg) DMSO for 300 mg/kg TC, 300 mg/kg EU, and 300 mg/kg CAR; and 12.68 mM (or 900 mg/kg) DMSO for 500 mg/kg CAR, respectively. The molar concentrations were calculated based on PDA/DMSO injected over body volumes (based on the average body density of 1.1 g/mL^16^).

A total of ten parameters were observed as follows: (1) inflammation of injection site [bump/raise], (2) eating/drinking behavior, (3) levels of activities, (4) overall physical appearance [grooming/fur], (5) body weight [Days − 2, 0, 2 and 4], and (6) foot/leg function/appearance. Mice were euthanized on the morning of Day 4 with CO_2_ and were necropsied subsequently. Upon dissection, additional parameters were observed: (7) inflammation/necrosis of the muscles of the injected legs by visual examination, (8) measurements of the hind legs at the proximal cranial thigh muscles [approximate location of injection], (9) weight of the liver, and (10) weights of the kidneys.

For treatments, a 40% stock solution (w/v) of each PDA was made in DMSO. The working dilution was made with PBS immediately before injection. The injectable was an emulsion and was fully mixed before administration into muscles of the right hind limb. The molar concentrations were calculated based on PDA/DMSO injected over body volumes (based on the average body density of 1.1 g/mL^16^). The control mice received the same percentage of DMSO as in the corresponding treated mice.

On the morning of Day 4, mice were weighed and euthanized with carbon dioxide inhalation. Animals were dissected for the weight of liver and kidney. The skin of both hind legs was removed and the color, swelling/necrosis of muscles were compared. The legs were disarticulated from the axial skeleton at the level of the coxofemoral joint. The widths of the legs at this level, including the proximal cranial thigh muscles (quadriceps femoris m. and iliopsoas m.) were measured. This was the approximate location of the injection.

### Animal scoring system

The scoring system of eating, activity, and appearance was adopted and modified from others ^17,18^ (Table 2). We also developed a scoring system based on appearance/functionality or abnormality of the foot/leg after injection (Table 2). During each observation, mice were scored individually on a scale of 0–3 for each parameter in Table 2. A score of 0 indicated normalcy whereas a score of 3 indicated not eating/drinking, severe dehydration, or foot/leg problem. At any observation point, a mouse would be humanely euthanized if the following score was received: a single score of 3 in any of the categories of eating/drinking, activities, or appearance; a combined score of ≥ 10; a body condition score of 1 or 5, or weight reduction of > 20%.

**Table 2:** Scoring systems for eating/drinking, physical activities, physical appearance, and foot/leg function/appearance.

Clinical score	Eating/drinking	Physical activities	Physical appearance	Foot/leg function
0	Drinking and eating well	Moves quickly around cage, frequently stands at sides, close to cage-mates	Normal	Unaffected
1	Change in eating or drinking habit	Reduced movements, little/no investigation of surroundings, seeks shelter, prefers cage corner, moves around cage when gently handled	Ruffled fur	Closed digits, walking normally
2	Inappetence	No movements around cage, moves slightly when gently handled, isolated from cage-mates	Weeping eyes	Closed digits, no grip
3	No eating or drinking, severely dehydrated	No movement by gentle handling, unable to right self within 30 s after being placed on back	Weeping/closed eyes, urine staining, difficulty defecating	Closed digits, no grip, dragging leg, foot flipped (tarsal extension, plantar side up)

**Figure 2:**
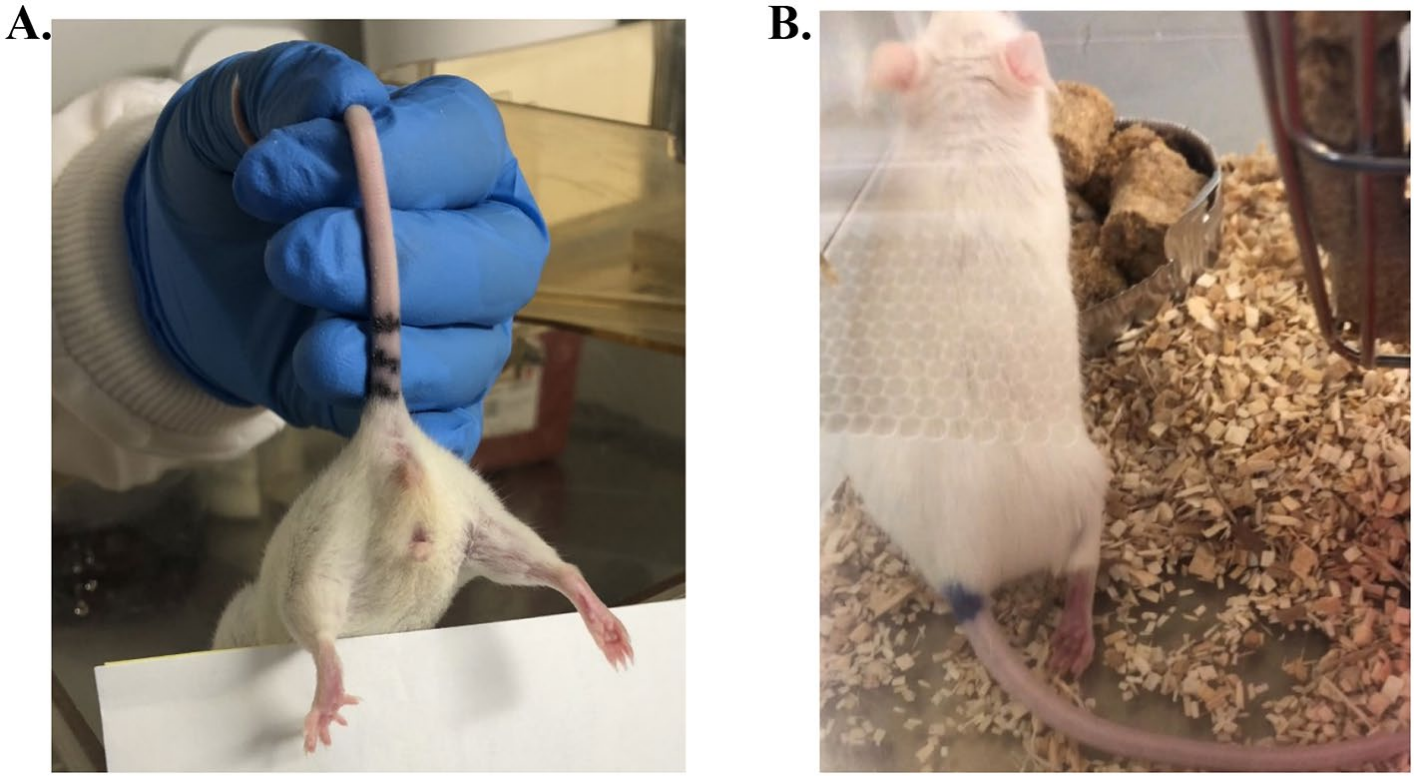
Representative images of mice with (**A**) a foot of closed digits, or (**B**) tarsal extension and dragging foot on the leg receiving injection. Animals here received scores of (**A**) 1 and (**B**) 3 for the foot/leg function parameter.

All scoring systems were adapted from^17,18^ with the exception for foot/leg scores which were developed in this study (see Fig. 2).

### Statistical analysis

Inflammation of the injection site (bump/raise), body weight (Days − 2, 0, 2, and 4), and weights of the liver and kidneys between treated mice and their respective controls were analyzed using unpaired two-samples t-test in R software^19^. Differences were considered significant when *P* < 0.05. Ordered scores (0, 1, 2, 3) for foot/leg and behavior over time, fitted to a logistic or probit regression model, were analyzed by an ordered logistic regression^20^ in R software^19^, using polr() function from the MASS package. *P*-value was calculated using cftest function from the multcomp package^21^. One-way ANOVA was used to determine the effect of PDA on leg muscle measurements; sex was used as a covariate.

## Results

### Effects of PDAs on external, pre-necropsy parameters

External observations included mortality, body weight, eating/drinking, physical activity, appearance, injection site, and foot/leg functionality. No mortality was observed in any of the control, TC, or EU groups. However, CAR exhibited more acute toxicity in males than females (Table 3). A total of three of the eight male mice receiving CAR died or were removed from the study. A male mouse from the 300 mg/kg CAR group was found dead on the evening of Day 0 (12 h after treatment). The mouse did not exhibit any behavior/physical abnormalities prior to death, but the deceased male was found to have facial wounds and was missing both eyes. These wounds could have occurred post-mortem, or aggression might have occurred prior to his death. However, we did not observe aggressive behavior in our twice-daily check-ins. In the 500 mg/kg CAR group, a second male mouse was euthanized on the morning of Day 1 (24 h after treatment) for receiving eating and appearance scores that exceeded the study’s humane endpoints. Finally, a third male in the 500 mg/kg group died on Day 2 (or 48 h after treatment). No signs of aggression were seen in these two males.

**Table 3:** Mortality in CAR treatment groups (dead/total). No mortality was found in the corresponding DMSO controls.

CAR levels	0	300 mg/kg	500 mg/kg
Female	0/4	0/4	0/4
Male	0/4	1/4	2/4

The average body weight for all control mice was 30.22 ± 4.33 and 32.75 ± 4.61 g, before and after the experiment, respectively. No significant differences in body weight were seen between any treated groups and their respective controls at any time of data collection on Days 0, 2, and 4 (data not shown).

No significant differences were observed between any treated groups and their corresponding controls in eating/drinking, activity, appearance, and foot/leg scores. Minor behavioral changes, such as reduced movements and ruffled fur (lack of grooming), however, were observed within an hour in one male from each of the 300 and 500 mg/kg TC treated groups (Table 4). A female mouse was also observed to have minor reduced activities 36 h after injection with 300 mg/kg TC (data not shown). All animals returned to pre-injection baseline parameters by the next observation point, either within 1 h (Table 4) or within 12 h (data not shown). Similarly, in the 500 mg/kg CAR group, reduced eating/drinking and/or physical activity/ruffled fur (appearance) were observed in a total of three males, at different observation times, within the first 12 h of injection (Table 4). The reduced eating/activity in two males lasted up to 36 h after receiving CAR. Three males injected with CAR received scores of > 0 in two or three categories, and of these, one died subsequently and the other was euthanized. CAR-induced effects seemed more severe, latent, and prolonged than those induced by TC. None of the EU treated animal received behavior/physical scores greater than 0.

**Table 4:** The averaged behavior scores and the number of animals receiving a score of > 0 during the first 12 h of TC and CAR treatments. No animals treated with EU received scores of > 0.

Treatment	1 h post-injection			12 h post-injection		
	Eating	Activity	Appearance	Eating	Activity	Appearance
Control	0	0	0	0	0	0
TC 300 mg/kg	0	0.125 (1/8)	0.125 (1/8)	0	0	0
500 mg/kg	0	0.125 (1/8)	0.125 (1/8)	0	0	0
Control	0	0	0	0	0	0
CAR 300 mg/kg	0	0	0	0	0	0
500 mg/kg	0	0.125 (1/8)	0.125 (1/8)	0.44 (2/8)	0.31 (2/8)	0.125 (1/8)

**Figure 3:**
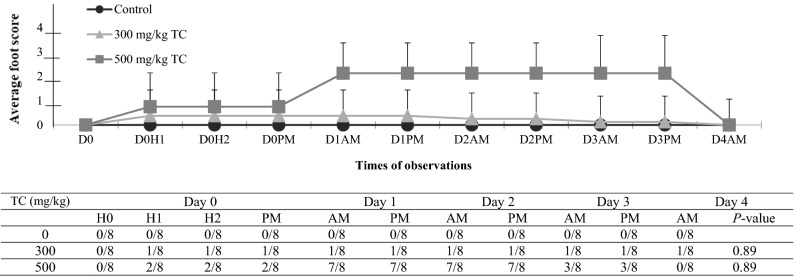
The averaged foot/leg scores (means ± SD; upper panel) and number of mice with scores of > 0 (lower panel) in controls and TC treated mice. D and H: day and hour post-injection. Scoring system: 0 = normal; 1 = closed digits but walked well; 2 = closed digits, no grip; 3 = closed digits, no grip, dragging leg, foot flipped.

**Figure 4:**
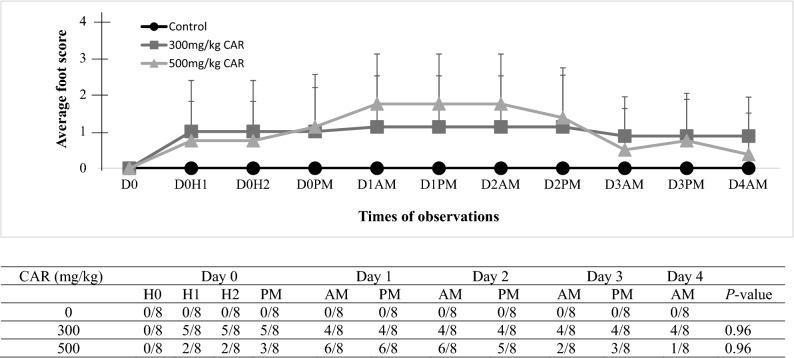
The averaged foot/leg scores (means ± SD; upper panel) and number of mice with scores of > 0 (lower panel) in controls and CAR treated mice. D and H: day and hour post-injection. Scoring system: 0 = normal; 1 = closed digits but walked well; 2 = closed digits, no grip; 3 = closed digits, no grip, dragging leg, foot flipped.

**Figure 5:**
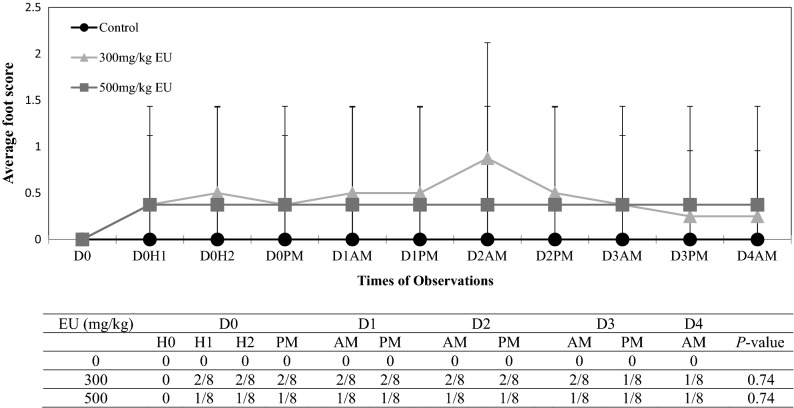
The averaged foot/leg scores (means ± SD; upper panel) and number of mice with scores of > 0 (lower panel) in controls and EU treated mice. D and H: day and hour post-injection. Scoring system: 0 = normal; 1 = closed digits but walked well; 2 = closed digits, no grip; 3 = closed digits, no grip, dragging leg, foot flipped.

No bumps or swelling (local inflammation) at the injection sites were observed during the entire experimental period. The observation of digit-closing and foot-flipping was not expected, but was carefully recorded, and a new scoring system was subsequently developed (Table 2, Fig. 2). Although various degrees of foot abnormalities were seen after injections of TC (Fig. 3), CAR (Fig. 4), or EU (Fig. 5), none of the foot scores in any of the treated groups were significantly different from controls. Overall, higher PDA doses affected more mice and were correlated with eliciting higher foot scores. Among the three PDAs used, TC elicited the greatest number of animals with foot/leg anomalies, seven out of eight during Days 1 and 2 at 500 mg/kg. This is followed by CAR with six out of eight in Day 1 at 500 mg/kg. EU had the least effect on foot scores, two out of eight animals. Most changes in the foot/leg occurred within the first hour of injection and, in the majority of cases, foot/leg functionality returned to normal or subsided by the end of the observation period (Day 4).

### Effects of PDAs on internal, post-necropsy parameters

**Figure 6:**
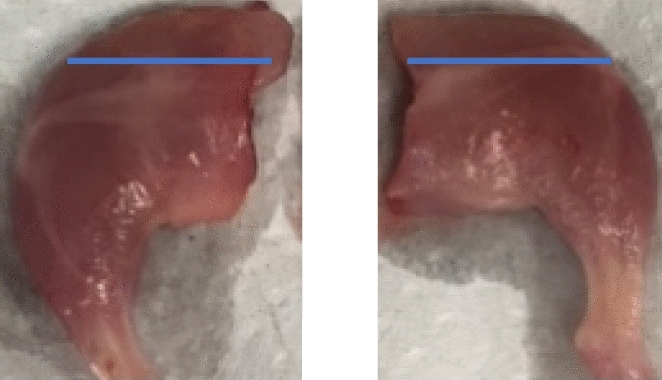
A representative necropsy image of muscles of the injected (right) vs. control (left) hind legs. The blue lines indicate where the cranial leg measurements were taken.

Internal observations include leg muscles, liver and kidneys. The gross characteristics of the muscles of the entire injected legs were compared to those of the non-injected counterparts (Fig. 6). No gross signs of inflammation, necrosis, generalized discoloration, or edema were observed in any animal, PDA-treated or controls.

Overall, there is no significant difference in PDA injected leg muscle measurements compared to their non-injected counterparts in any groups (*P* > 0.05). Data from all groups were then combined, and the non-injected and injected legs measured 1.45 ± 0.23 cm and 1.54 ± 0.27 cm, respectively (Fig. 6). The relative liver weights of mice treated with both 300 and 500 mg/kg EU, however, were significantly higher than that of the corresponding controls (*P* < 0.05) (Table 5). Similarly, the relative weights of the kidneys were significantly different between 300 mg/kg CAR-treated and corresponding controls (*P* < 0.001) (Table 5). Although these changes were statistically significant, they were ≤ 1% compared to corresponding controls. It is unlikely that these observations are of any biological significance.

**Table 5:** Total mouse body weights, quantitative organ weights (g), and relative organ weights (%) of the liver and kidneys in control and EU- or CAR-treated mice.

Treatments	Body weight (g)	Liver (g)	Kidney (g)	Relative liver (%)	Relative kidney (%)
Control	27.88 ± 2.30	1.96 ± 0.32	0.46 ± 0.12	7.01 ± 0.72	1.65 ± 0.32
EU (300 mg/kg)	31.25 ± 3.11	2.44 ± 0.40	0.49 ± 0.10	7.76 ± 0.63*	1.56 ± 0.25
Control	31.62 ± 5.42	2.20 ± 0.65	0.54 ± 0.18	6.84 ± 1.03	1.67 ± 0.36
EU (500 mg/kg)	32.12 ± 5.96	2.55 ± 0.67	0.58 ± 0.20	7.84 ± 0.72*	1.75 ± 0.35
Control	34.0 ± 5.66	2.53 ± 0.76	0.63 ± 0.19	7.29 ± 1.08	1.80 ± 0.31
CAR (300 mg/kg)	32.25 ± 5.68	2.59 ± 0.55	0.81 ± 0.21	7.99 ± 0.54	2.50 ± 0.35**

* and **: significantly different from the corresponding controls at *P* < 0.05 and *P* < 0.001 levels, respectively.

## Discussion

Antimicrobial effects of PDAs have been widely demonstrated in vitro such as in bacterial culture studies. In vivo studies have shown that PDAs as feed additives significantly reduced *E. coli* colonization in the urinary tract infection murine model^22^ and cecal populations of *S. enteritidis* and *C. jejuni* in chickens^7^. A recent study found that oral-gavaged CAR was associated with increased survival of mice infected with *Klebsiella pneumoniae* carbapenemase, and significantly reduced bacterial load in peritoneal lavage ^23^. Additionally, PDA effects after parenteral injection have been examined, however, they have only been tested through ip, sc, or iv administration and mainly for toxicity studies. The use of PDAs as antimicrobials by im injection has not been documented in the literature. This study is the first to determine the toxicity of *trans*-cinnamaldehyde, eugenol, and carvacrol after im administration using the murine model. With ten clinical, behavioral, or post-mortem parameters, each observed for three PDAs administered at two different doses (together with their respective DMSO controls), a total 81 data points were collected. Statistically significant differences were only found in the higher relative liver weight (300 and 500 mg/kg EU group), and the higher relative kidney weight (300 mg/kg CAR group). The most severe effect was death of three males out of 16 CAR-treated mice. The second most severe consequence was that a high percentage of mice had foot/leg anomalies, especially with the higher levels of TC and CAR. However most, if not all, mice recovered by the end of the study or 4 days post-injection. Other changes in behaviors and activities were minor and only occurred in a few mice. All animals returned to baseline parameters by the end of the study. These data suggest the overall tolerance of im-injected PDAs.

Carvacrol is generally regarded as safe for consumption and has been used in low doses as a flavoring constituent and natural preservative for foods. For potentially effective therapeutic applications, higher doses may be needed. Toxicity studies of carvacrol are limited^24^. In the mouse, it was estimated that the median lethal doses for iv and ip administration of CAR were 80.0 and 73.3 mg/kg of body weight, respectively^12^. The exact mechanism of the toxicity could be a combination of carvacrol’s effects on cytotoxicity, mitochondria, and intracellular calcium homeostasis^24^. While it was not the purpose here to establish an LD50 for im administration of carvacrol, three of the eight male mice died with 300–500 mg/kg im injection, while no females exhibited CAR-induced toxicity, suggests that there is a significant sex-linked difference in CAR-induced toxicity. It is unclear why males responded more strongly than females to the higher doses of CAR. This observation could be a co-incident due to the small numbers of animals used. To accommodate the social nature of mice^25^, we group-housed the males and did not observe aggression in any group during acclimation or in any treated groups other than CAR. Therefore, the male deaths (apart from the one in the 300 mg/kg CAR group) were unlikely due to male-male aggression. We did not find any prior reports in the literature that documented sex differences in acute toxicity from carvacrol or other PDAs. While not related to our findings, the only report of sexual difference in response to essential oil or PDA treatments was in rats^26^. Males and females responded differently upon persistent nociceptive input of 5% formalin subcutaneously injected to the hind paw and inhaling aroma from citrus lemon^26^. Our observation of sex-linked toxicity is therefore the first documented example of carvacrol toxicity.

No gross indications of local inflammation were observed throughout the experimental period, and necrosis was not seen at post-mortem dissection. These suggest that PDAs were quickly absorbed and the doses injected did not cause local tissue damage. The highest PDA concentration that can be dissolved in the DMSO was 40% (or 2.66 M CAR; 2.44 M EU and 3.03 M TC). When working concentrations of PDAs were prepared by dilution with PBS, the PDA/DMSO/PBS mix became an emulsion. This, however, did not seem to have affected the absorption of PDAs because no local accumulation was found and systemic effects, such as the minor behavior change, were quickly observed. The only local effect was the partial loss of foot function and abnormal foot appearance, which occurred in all PDA-treated groups. Constituents of essential oils, such as carvacrol and eugenol, have been reported to accumulate in the neuronal membrane, thus causing steric blocking of embedded proteins, including ion channels^27,28^. This blocking causes a change in the physical properties of the neuron membrane, notably a modification of its ion permeability. Inhibition of the inflow of calcium ion inflow suppresses the generation of action potentials, thus causing local anesthetic effects. Similarly, Moreira-Lobo et al*.* also reported that eugenol blocked rat sciatic nerve compound action potentials, likely by acting on the voltage dependent Na^+^ channels, consequently reducing neuronal excitability^29^. Similar occurrences have been found in carvacrol in blocking excitability in the sciatic^30^ and peripheral nerve fibers, and a reduction of neuronal excitability^27,31^. The interference of neuronal excitability by im-injected PDAs was observed in our study as local muscle relaxation, inability of the feet to grip and, in severe cases, dragging of the foot. As PDAs were metabolized, these symptoms dissipated, and likely no permanent nerve damage was induced at the levels tested.

Carvacrol^32^, eugenol^33^, and *trans*-cinnamaldehyde^34^ are all metabolized in the liver and excreted in the kidney within 24 h of administration. Our observation of higher relatively liver and kidney weights from 500 mg/kg EU and 300 mg/kg CAR groups were similar to those reported in both males and female rats^26^ when a derivative of cinnamaldehyde, α-amyl cinnamaldehyde, was fed for 14 weeks. Previous studies indicated that relative kidney weight also increased in PDA-treated male rats^35^. Previous observations in collaboration with our findings suggest possible burden on both the liver and kidneys in the deactivation and excretion of administered PDAs, although the effects were very minor (≤ 1% changes). These side effects are different, however, than the reported liver injury induced by antibiotic useage^36^.

## Conclusion

Three plant-derived antimicrobials (PDAs) were tested for toxicity in mice after intramuscular administration and were measured using ten clinical/behavioral/post-mortem parameters. This study provides the first report of carvacrol, eugenol, and *trans*-cinnamaldehyde-induced toxicity when administered im. Overall, little to moderate toxicity was observed when PDAs were injected intramuscularly at the doses used, with minor side effects improving over the span of 4 days. Only carvacrol appeared to induce acute toxicity, and only in male mice. The data support the idea that certain PDAs could be used as therapeutic antimicrobials, if administered intramuscularly.
